# Influence of genotyping error in linkage mapping for complex traits – an analytic study

**DOI:** 10.1186/1471-2156-9-57

**Published:** 2008-08-25

**Authors:** Jérémie JP Lebrec, Hein Putter, Jeanine J Houwing-Duistermaat, Hans C van Houwelingen

**Affiliations:** 1 Department of Medical Statistics and Bioinformatics, Leiden University Medical Center, Postzone S-05-P, PO Box 9600 2300 RC Leiden, The Netherlands

## Abstract

**Background:**

Despite the current trend towards large epidemiological studies of unrelated individuals, linkage studies in families are still thoroughly being utilized as tools for disease gene mapping. The use of the single-nucleotide-polymorphisms (SNP) array technology in genotyping of family data has the potential to provide more informative linkage data. Nevertheless, SNP array data are not immune to genotyping error which, as has been suggested in the past, could dramatically affect the evidence for linkage especially in selective designs such as affected sib pair (ASP) designs. The influence of genotyping error on selective designs for continuous traits has not been assessed yet.

**Results:**

We use the identity-by-descent (IBD) regression-based paradigm for linkage testing to analytically quantify the effect of simple genotyping error models under specific selection schemes for sibling pairs. We show, for example, that in extremely concordant (EC) designs, genotyping error leads to decreased power whereas it leads to increased type I error in extremely discordant (ED) designs. Perhaps surprisingly, the effect of genotyping error on inference is most severe in designs where selection is least extreme. We suggest a genomic control for genotyping errors via a simple modification of the intercept in the regression for linkage.

**Conclusion:**

This study extends earlier findings: genotyping error can substantially affect type I error and power in selective designs for continuous traits. Designs involving both EC and ED sib pairs are fairly immune to genotyping error. When those designs are not feasible the simple genomic control strategy that we suggest offers the potential to deliver more robust inference, especially if genotyping is carried out by SNP array technology.

## Background

Linkage analysis of family data have been extensively used in the past in the search for genetic determinants. Nowadays, investigators favor large epidemiological studies of unrelated individuals, however several family datasets are currently being re-analyzed and/or pooled (e.g. [1]). The persistance of interest for linkage is partly triggered by the advent of single-nucleotide-polymorphisms (SNP) array genotyping technology in the field, indeed SNP arrays hold the promise of more reliable linkage maps [2,3]. Although less prone to genotyping error than microsatellites when viewed as singlepoint markers, SNP arrays heavily rely on multipoint algorithms for accurate determination of the identical by descent (IBD) status of alleles. The gain in singlepoint reliability might therefore be annihilated by the propagation of errors across the many SNPs required to infer IBD status.

In the search for genetic determinants of complex traits by linkage, the use of selective designs appears to be an efficient way to gain adequate power for detection of typically small gene effects. A few authors have shown by simulation that the impact of genotyping error on evidence for linkage could be particularly severe in affected sib-pair (ASP) designs [4-6], virtually masking most of the evidence for linkage. The impact of error on quantitative traits appears to be less dramatic in random samples, however it is unclear whether the same dramatic power losses hold in selected samples.

A method of choice is now emerging for the analysis of quantitative traits arising from selected sib pairs. This method is essentially a regression through the origin of excess identical by descent (IBD) sharing on a function of the trait value, whose slope is an estimate of the linkage parameter. It was first proposed by Sham et al. [7] and turns out to be equivalent to a score test [8]. In a numerical comparison of methods for selected samples, Skatkiewicz et al. [9] and Cuenco et al. [10] showed that this method had good properties in finite samples for extreme proband ascertained sib-pair and discordant sib-pair designs. By use of simple genotyping error models (*population frequency error model *and *false homozygosity model*), we show analytically what effects such error generating processes (occurring at rate ϵ per sib pair) induce for an idealized fully informative marker. It is shown that it results in a reduction of the slope estimate (i.e. of the estimated linkage parameter) by a factor 1 - $\frac{\epsilon }{2}$ whether sib pairs are selected or not. Since the genotyping error rate ϵ is typically small, the previous effect on the linkage test is minimal. In addition to this slope effect, the regression's intercept is modified and this may have a much more sizable effect on the test for linkage depending on the sampling scheme used to select sib pairs. Surprisingly, this simple result allows us to predict that in extremely concordant (EC) sib pairs designs and in ASP designs, the effect of genotyping error will be milder as the selection becomes more extreme. In extreme discordant (ED) designs, the effect can in theory be either increased type I error or decreased power depending on the definition of discordance, the genotyping error rate and the true linkage effect; in practice however, for small quantitative trait locus (QTL) effects, the result will be an increased type I error. We argue that the basic error generating mechanisms assumed provide reasonable approximations of real-life situations. In the next section, we first describe some common error-generating processes and quantify their effect on IBD sharing in an idealized situation where marker information is complete. We then briefly sketch the inverse regression approach to linkage, we show analytically what the effect of genotyping error is on this regression and quantify the subsequent bias, power and type I error in common selective designs. We argue that under certain assumptions regarding the error model, one can easily implement a linkage test that incorporates a genomic control for genotyping error. Finally, we discuss some assumptions made in our study and the practical relevance of our findings. In particular, we argue that our results generalize to situations where marker information is incomplete and that the smaller error rates observed in SNP chip array compared to microsatellites offer no protection against bias in analysis.

## Results

### Genotyping error models

We consider two mechanisms for the generation of errors in marker data, namely the *population frequency error model *and the *false homozygosity model*. In those two models, we consider a single marker with *m *alleles and further assume that a maximum of one allelic error per sib pair can be made and that this happens with probability ϵ. This restriction to 'one error per sib pair' is just a first order approximation, for small ϵ, of a process where all four alleles would be allowed to be independently erroneous and does not restrict the generalizability of our results.

The *population frequency error model *re-assigns the erroneous allele (chosen at random among the four forming the sib-pair genotype) to one of the possible *m *alleles with probability equal to population allele frequency. One mathematical advantage of this model is that the marginal distribution of alleles and genotypes is unaltered. The *false homozygosity model *keeps homozygotes unchanged but re-assigns heterozygotes to homozygotes with alleles equal to one of the two original alleles chosen according to probabilities proportional to population allele frequencies.

To our knowledge, *false homozygosity *is a common type of error: fairly rare alleles go un-reported in samples. The *population frequency error model *provides an approximation to a process whereby alleles are misread. Errors at the two alleles of a marker's genotype might be correlated, we do not consider this type of process in details here although the effect on linkage will be qualitatively the same as in the two other models. We refer the reader to Sobel et al. [11] for a detailed exposé on genotyping error mechanisms. Note that the two models that we have chosen have been used in the past in order to identify potential genotyping errors [4,11].

### Impact on IBD sharing

Let's denote by *π *the proportion of alleles shared identical by descent (IBD) at a certain locus by two siblings. Tests for linkage are based on the IBD sharing distribution and although errors as described earlier are made at the genotype level (*G *is read as *G*^ϵ^), the effect of errors on linkage will be entirely mediated via the distortion of the IBD distribution (the true IBD status *π *of two siblings may be incorrectly inferred as *π*^ϵ^). We are therefore interested in deriving the probability distribution **P**(*π*^ϵ^|*π*), this is done by conditioning on both the true and observed genotypes as follows:

$$P(\pi^{\epsilon }|\pi )=\sum_{G^{\epsilon }}P(\pi^{\epsilon }|G^{\epsilon })\sum_{G}P(G^{\epsilon }|G)\;P(G|\pi ).$$

Let us consider the case of complete information. This can be conceptualized by means of an idealized marker whose number of alleles is infinite, in particular identity by state (IBS) status is equivalent to IBD status. The unordered genotypes of a sib pair can be partitioned into seven exclusive classes denoted *ii*/*ii*, *ii*/*ij*, *ii*/*jj*, *ii*/*jk*, *ij*/*ij*, *ij*/*ik *and *ij*/*kl *depending on the number of homozygous sibs in the pair and the number of distinct alleles in the sib-pair genotype. Sharing 0 alleles IBD corresponds to a sib-pair genotype of the *ij*/*kl *class, should an error occur according to the *population frequency error model *then one of the four alleles would be transformed into yet another type (since the number of alleles is infinite, the probability that the new allele is read as one of *i*, *j*, *k *or *l *tends to 0), therefore the sib pair genotype will remain in the *ij*/*kl *class and the observed IBD status *π*^ϵ ^will still be 0. For the same starting genotype, an error according to the *false homozygosity model *produces an *ii*/*jk *class and *π*^ϵ ^also equals 0 therefore **P**(*π*^ϵ ^= 0|*π *= 0) = 1 whatever the genotyping error mechanism considered previously. The same line of reasoning leads to **P**(*π*^ϵ ^= 0.5|*π *= 0.5) = 1 - $\frac{\epsilon }{2}$, **P**(*π*^ϵ ^= 0|*π *= 0.5) = $\frac{\epsilon }{2}$, **P**(*π*^ϵ ^= 1.0|*π *= 1.0) = 1 - ϵ, **P**(*π*^ϵ ^= 0.5|*π *= 1.0) = ϵ. Those results can be summarized by the transition matrix below, where the (*i*, *j*) element is equal to **P**(*π*^ϵ ^= (*j *- 1)/2|*π *= (*i *- 1)/2)

$$P(\pi^{\epsilon }|\pi )=\left(\begin{array}{ccc} 1 & 0 & 0\\ \frac{\epsilon }{2} & 1-\frac{\epsilon }{2} & 0\\ 0 & \epsilon & 1-\epsilon \end{array}\right).$$

The overall effect of genotyping error is thus to reduce the observed IBD sharing, indeed **E**(*π*^ϵ^|*π*) = (1 - ϵ/2)*π *and **E**(*π*^ϵ^) = $\frac{1}{2}$ - ϵ/4 while the variance is practically unchanged since $\mathrm{var}(\pi^{\epsilon })=\frac{1}{8}-\frac{1}{16}\epsilon^{2}$. In selected samples of extremely concordant sib pairs (EC) where linkage is evidenced by an excess in IBD sharing, it therefore seems logical to expect a decrease in power. Conversely, in selected samples of extremely discordant sib pairs (ED) where linkage is evidenced by a reduction in IBD sharing, the test might lead to increased type I error. In the next subsection, we formally quantify this bias in selective samples schemes for quantitative traits under the usual assumption of a normal variance components model.

### Impact on linkage testing

#### Regression-based linkage testing

We assume that the sib pair phenotypic data **x **= (*x*_1_, *x*_2_)*' *have been adjusted for any relevant covariates (e.g. sex, age, country, ...) and have been standardized so that the (known) population mean, variance and sib-sib correlation are 0, 1 and *ρ *respectively. Under the additive variance components model, **x **given IBD information *p *follows a bivariate normal distribution with zero mean and variance-covariance matrix given by

$$\left(\begin{array}{cc} 1 & \gamma (\pi -\frac{1}{2})+\rho \\ \gamma (\pi -\frac{1}{2})+\rho & 1 \end{array}\right),$$

where *γ *≥ 0 denotes the proportion of total variance explained by the putative locus. Under this model, an optimal testing strategy first advocated in [7] (and sometimes referred to as the optimal Haseman-Elston regression) is to regress (through the origin) excess IBD sharing *π *- $\frac{1}{2}$ on the following *C *function of the trait values:

(1) $$C(x_{1},x_{2},\rho )=\frac{\left(1+\rho^{2})x_{1}x_{2}-\rho (x_{1}^{2}+x_{2}^{2})+\rho (1-\rho^{2}\right)}{{\left(1-\rho^{2}\right)}^{2}}.$$

This test turns out to be a score test for the linkage parameter *γ *[8] and is based upon the following approximate relation which is valid for small locus effects [12]:

(2) $$E(\pi -\frac{1}{2}|x,\gamma )=\frac{\gamma }{8}C(x,\rho ),$$

where $\frac{1}{8}$ = var_0_(*π*). In a set of sibships indexed by *i*, an efficient estimate of the linkage parameter *γ *is $\hat{\gamma }=8\frac{\sum_{i}(\pi_{i}-\frac{1}{2})C_{i}}{\sum_{i}C_{i}^{2}}$. It is approximately unbiased **E**($\hat{\gamma }$) = *γ *and has variance var_0_($\hat{\gamma }$) = 1/I where $I=\frac{1}{8}\sum_{i}C_{i}^{2}$ is the corresponding Fisher's information. The test statistic is given by $\hat{\gamma }\sqrt{I}$, it is one-sided, only positive values being regarded as evidence for linkage. For small QTL effects, power of this test can be computed as Φ (Φ^-1^(*α*) + *γ*I^1/2^). Fisher's information I, which depends on sample size and study design, therefore controls power. In the design phase of a study, I should be used as a criterion to differentiate between alternative designs rather than sample size only [12,13].

#### Impact of genotyping error on regression

By conditioning on the true IBD sharing values, we can compute **P**(*π*^ϵ^|**x**, *γ*, ϵ) = ∑_*π*_**P**(*π*^ϵ^|*π*) **P**(*π*|**x**, *γ*), using the transition probabilities **P**(*π*^ϵ^|*π*) derived earlier, while the **P**(*π*|**x**, *γ*)'s are given in [12]. This permits computation of the new regression line in presence of genotyping error as

(3) $$E(\pi^{\epsilon }-\frac{1}{2}|x,\gamma ,\epsilon )=-\frac{\epsilon }{4}+(1-\frac{\epsilon }{2})\frac{\gamma }{8}C(x,\rho ).$$

As mentioned earlier, the corresponding variance under the null hypothesis is only slightly altered. The effect of genotyping error is thus to shrink the regression line by a factor 1 - $\frac{\epsilon }{2}$ and to shift the intercept by -$\frac{\epsilon }{4}$. If we ignore genotyping error i.e. we estimate *γ *using ${\hat{\gamma }}^{\epsilon }=8\frac{\sum_{i}(\pi_{i}-\frac{1}{2})C_{i}}{\sum_{i}C_{i}^{2}}$, this results in a biased estimator $\text{bias}({\hat{\gamma }}^{\epsilon })=E({\hat{\gamma }}^{\epsilon })-\gamma =-\epsilon \left(\frac{\gamma }{2}+2A\right)$ with $A=\frac{\sum_{i}C_{i}}{{\sum C}_{i}^{2}}=\frac{\bar{C}}{\bar{C^{2}}}$. The resulting testing statistic ${\hat{\gamma }}^{\epsilon }I^{1/2}$ would then have power equal to

(4) $$\Phi \left(\Phi^{-1}(\alpha )+\gamma I^{1/2}+\text{bias}({\hat{\gamma }}^{\epsilon })I^{1/2}\right).$$

Note that taking *γ *= 0 in this formula gives the type I error rate. Since I increases with sample size, the impact of genotyping error on both power and type I error will be larger as the sample size increases. In terms of Y versus X regression, the intuition is that the regression through the origin is not affected by a general shift in the Y-variable (IBD sharing) if the X-variable (*C *variable) has average 0, or takes values far away from 0. The further away the X-variable *C *is from 0, the smaller *A*, hence the smaller the bias.

#### Bias and impact on power and type I error

Since $\text{bias}({\hat{\gamma }}^{\epsilon })=-\epsilon \left(\frac{\gamma }{2}+2A\right)$ and *γ *is typically small, the distortion of the usual linkage test in presence of genotyping error heavily depends on the design-specific quantity $A=\bar{C}/\bar{C^{2}}$. Unfortunately, there is little intuition about the distribution of *C *(hence about the distribution of *A*) in the whole population or in a selected sample. Nevertheless, Monte Carlo simulations can be used to determine the characteristics of the *C *and *A *distributions in the whole population or for a specific ascertainment scheme. In random samples and under the variance components model, *C *is a score function hence **E**(*C*) = 0 therefore its sample estimate $\bar{C}$ will be close to 0; one can also check that its distribution is negatively skewed (unless *ρ *= 0). The result is that the bias will be small for random samples. The same finding would hold for any ascertainment scheme where $\bar{C}$ = 0. An optimal selection scheme [12] that would select sib pairs based on Fisher's information I (i.e. such that |*C*| ≥ *C*_0_) does not warrant that $\bar{C}$ = 0 because of the skewness of *C*. In EC designs (both siblings have trait values either larger than a positive threshold or smaller than a negative threshold), $\bar{C}$ tends to be positive while it tends to be negative in ED designs (one sibling's trait value is larger than a positive threshold while the other sibling's trait value is smaller than a negative threshold), the linkage test will therefore have reduced power in EC designs and increased type I error in ED designs.

In the left-hand side of Table 1, we have computed the values of *A *and $\bar{C}$ for the three selective schemes considered. The designs are indexed by the sib-sib correlation *ρ *and the degree of selection. One obvious way to correct for the shift in the intercept induced by genotyping error would be to leave the regression unconstrained, this would correct for most of the bias. Unfortunately, in selected designs where the variance of *C *is reduced, this results in a very inefficient estimator of the linkage parameter *γ*. The right-hand side of Table 1 displays the variance of the linkage parameter estimates in constrained (${\mathrm{var}}_{\text{con}}(\hat{\gamma })=1/\sum_{i}C_{i}^{2}$) and unconstrained (${\mathrm{var}}_{\text{uncon}}(\hat{\gamma })=1/\sum_{i}{\left(C_{i}-\bar{C}\right)}^{2}$) regressions. Efficiency losses of unconstrained versus constrained regressions in EC and ED designs are unacceptably large even for moderately extreme selection schemes.

**Table 1:** Bias in selective designs

		*A*			$\bar{C}$			var_con_			var_uncon_		
					
Selection	*ρ*	EC	ED	I	EC	ED	I	EC	ED	I	EC	ED	I
1%	0.1	0.27	-0.23	-0.07	3.45	-3.93	-1.61	0.08	0.06	0.04	1.13	0.68	0.05
	0.2	0.29	-0.21	-0.13	3.28	-4.25	-3.19	0.09	0.05	0.04	1.46	0.52	0.07
	0.3	0.30	-0.19	-0.15	3.15	-4.72	-4.63	0.10	0.04	0.03	1.82	0.38	0.10
	0.4	0.31	-0.17	-0.14	3.06	-5.29	-6.00	0.10	0.03	0.02	2.27	0.27	0.17
	0.5	0.32	-0.14	-0.12	3.01	-6.10	-7.44	0.11	0.02	0.02	2.38	0.18	0.23
	0.6	0.31	-0.12	-0.10	3.02	-7.33	-9.33	0.10	0.02	0.01	1.92	0.12	0.19
													
10%	0.1	0.47	-0.40	-0.06	1.71	-1.87	-0.40	0.28	0.22	0.14	1.48	0.88	0.14
	0.2	0.50	-0.36	-0.11	1.66	-1.99	-0.81	0.30	0.18	0.13	1.84	0.66	0.14
	0.3	0.52	-0.32	-0.14	1.64	-2.14	-1.30	0.32	0.15	0.11	2.20	0.48	0.13
	0.4	0.53	-0.28	-0.16	1.63	-2.35	-1.86	0.32	0.12	0.09	2.37	0.35	0.12
	0.5	0.52	-0.24	-0.17	1.64	-2.61	-2.61	0.31	0.09	0.06	2.05	0.23	0.11
	0.6	0.47	-0.19	-0.15	1.68	-3.01	-3.64	0.28	0.06	0.04	1.33	0.15	0.10
													
30%	0.1	0.65	-0.53	-0.04	0.96	-1.01	-0.11	0.68	0.52	0.31	1.80	1.12	0.32
	0.2	0.69	-0.46	-0.07	0.95	-1.03	-0.23	0.73	0.44	0.29	2.15	0.85	0.29
	0.3	0.71	-0.39	-0.09	0.96	-1.06	-0.36	0.74	0.37	0.25	2.33	0.63	0.26
	0.4	0.69	-0.32	-0.11	0.97	-1.13	-0.52	0.71	0.28	0.20	2.17	0.45	0.21
	0.5	0.62	-0.25	-0.11	1.00	-1.22	-0.73	0.62	0.21	0.15	1.64	0.30	0.16
	0.6	0.50	-0.19	-0.10	1.05	-1.35	-1.01	0.47	0.14	0.10	0.98	0.19	0.11

Left-hand side: Values of *A*, $\bar{C}$ quantities influencing the effect of genotyping error for a variety of selective designs indexed by degree of Selection and sib-sib trait correlation *ρ*) – Right-hand side: Comparison of efficiency in constrained and unconstrained regressions – See text for definitions of *A*, $\bar{C}$, var_con _and var_uncon_

In Table 2, we report the power and type I error for realistic genotyping error rates [14] equal to 0.005 and 0.01 for the same designs as in Table 2. The equivalent sample size used corresponds to samples with Fisher's information equal to 2500 which provides 90% power to detect a QTL explaining 10% of the total variance in absence of genotyping error (pointwise nominal error rate = 10^-4^). The most visible impact is on type I error rates in ED design which is up to 7 times its nominal value. The I design that combines EC and ED sib pairs appears to be fairly immune to genotyping error while EC designs do not incur power losses greater than 20%. Finally, those computations confirm the intuition expressed earlier that the effect of genotyping error is less severe in more extreme selection schemes.

**Table 2:** Impact of genotyping error (rate = ϵ) on type I error and power

Error rate ϵ	Selection	*ρ*	EC		ED		I	
					
			Power	Type I Error × 10^-4^	Power	Type I Error × 10^-4^	Power	Type I Error × 10^-4^
0.005	1%	0.1	0.87	0.6	0.92	1.6	0.90	1.1
		0.2	0.87	0.6	0.92	1.5	0.91	1.3
		0.3	0.87	0.5	0.91	1.5	0.91	1.3
		0.4	0.87	0.5	0.91	1.4	0.91	1.3
		0.5	0.87	0.5	0.91	1.4	0.91	1.3
		0.6	0.87	0.5	0.91	1.3	0.91	1.2
	10%	0.1	0.85	0.4	0.93	2.2	0.90	1.1
		0.2	0.85	0.4	0.93	2.0	0.91	1.2
		0.3	0.84	0.3	0.92	1.9	0.91	1.3
		0.4	0.84	0.3	0.92	1.7	0.91	1.4
		0.5	0.84	0.3	0.92	1.6	0.91	1.4
		0.6	0.85	0.4	0.91	1.5	0.91	1.3
	30%	0.1	0.83	0.3	0.94	2.8	0.90	1.1
		0.2	0.82	0.2	0.93	2.4	0.90	1.1
		0.3	0.82	0.2	0.93	2.1	0.91	1.2
		0.4	0.82	0.2	0.92	1.9	0.91	1.2
		0.5	0.83	0.3	0.92	1.6	0.91	1.2
		0.6	0.85	0.4	0.91	1.5	0.91	1.2
								
0.01	1%	0.1	0.84	0.3	0.93	2.4	0.91	1.3
		0.2	0.83	0.3	0.93	2.2	0.92	1.7
		0.3	0.83	0.3	0.93	2.1	0.92	1.8
		0.4	0.83	0.3	0.92	1.9	0.92	1.7
		0.5	0.83	0.3	0.92	1.7	0.92	1.6
		0.6	0.83	0.3	0.92	1.6	0.91	1.5
	10%	0.1	0.78	0.1	0.95	4.5	0.91	1.3
		0.2	0.78	0.1	0.95	3.9	0.91	1.5
		0.3	0.77	0.1	0.94	3.4	0.92	1.7
		0.4	0.77	0.1	0.94	2.9	0.92	1.9
		0.5	0.77	0.1	0.93	2.5	0.92	1.9
		0.6	0.78	0.1	0.93	2.1	0.92	1.8
	30%	0.1	0.73	0.1	0.96	7.1	0.90	1.2
		0.2	0.71	0.1	0.96	5.6	0.91	1.3
		0.3	0.71	0.0	0.95	4.4	0.91	1.4
		0.4	0.71	0.1	0.94	3.4	0.91	1.5
		0.5	0.74	0.1	0.93	2.6	0.91	1.5
		0.6	0.78	0.1	0.93	2.1	0.91	1.5

Impact of genotyping error (rate = ϵ) on power and type I error of linkage test in selective designs (indexed by degree of Selection and sib-sib trait correlation *ρ*) – Nominal error rate = 10^-4^, QTL effect *γ *= 0.1 and sample size equivalent to a Fisher's information = 2500 in each design (provides 90% power in absence of genotyping error)

### Genomic control for genotyping error

As we have seen in previous sections, the main effect of genotyping error is to modify the intercept in the regression used to test for linkage. Although an unconstrained regression would correct most of the bias due to genotyping error, the inefficiency of this strategy makes it impractical. In order to obtain an efficient and robust inference, it therefore seems natural to try and constrain the regression through its correct origin *a*. In this section, we propose a completely data-driven strategy for doing this.

At any position, the sample mean IBD sharing has variance 1/8*n *where *n *is the number of sib pairs available. If we knew that the position is unlinked or if the sample of sib pairs was random then the deviation of this mean from $\frac{1}{2}$ would provide an estimate of the intercept *a *in the linkage regression.

Unfortunately, detection of a position-specific intercept corresponding to typical error rates would require a sample size of order 10^4^, a number that is almost never reached in linkage studies. In order to obtain an intercept estimate $\hat{a}$ with sufficient precision, it is therefore essential to combine information across positions. The value of IBD sharing at positions outside of the neighborhood of influencing loci (those positions are subsequently referred to as unlinked) across the genome may serve as control in the test for linkage, this concept of genomic control has been used to make the analysis of association studies more robust [15].

Let's assume that the proportions of alleles shared IBD *π *is computed at a series of approximately regular positions indexed by *t *across the whole genome. Let *y*_*t *_be the sample mean (among families) excess IBD at position *t *i.e. $y_{t}\equiv \bar{\pi_{t}^{\epsilon }}-0.5$. Under the variance components model and for small QTL effect *γ*, equation (3) implies that

$$E(y_{t})≃\{\begin{array}{ll} a, & \text{if position }t\text{ is unlinked},\\ a+\frac{b}{8}\gamma \bar{C}, & \text{if position }t\text{ is linked}. \end{array}$$

In random samples or in any sample where $\bar{C}$ ≃ 0, taking the average of *y*_*t *_across positions provides an estimate of *a*. In selected samples, we can use a trimmed version of the mean of *y*, for example a 20%-trimmed mean of the (*y*_*t*_)_*t *_series (i.e. the mean of the *y*_*t *_values after removing the 20% lowest and and 20% highest values) will provide a robust genomic estimate $\hat{a}$ of *a*. Because *a *≤ 0 and $\bar{C}$ is positive and negative in EC designs and ED designs respectively, $\hat{a}$ could be refined by trimming off only the 20% highest and lowest *y*_*t *_values respectively before taking the mean. Of course, how much we trim is arbitrary but 20% can safely be taken as a conservative value for oligogenic traits (Indeed, a 3500 cM genome contains approximately 70 quasi-independent loci, so a 20% trimming of *y*_*t *_values discards 14 positions (including all active gene positions if less than 14 genes) from the sample used to estimate intercept *a*.). An ad-hoc implementation of the concept of genomic control is then to plug in the estimate of the intercept $\hat{a}$ into the linkage regression (3). Since most of the bias in the inference is due to the intercept mis-specification, the precise estimate obtained by pooling across the genome will eliminate it. The implicit assumption that we make in this genomic control approach is that the regression intercept is the same at all positions, this will be challenged in the next section.

## Discussion

Under two basic error models, we were able to predict quantitatively the consequences of genotyping error on inference in linkage analysis. In the idealized situation of complete IBD information, both error models have the same impact on linkage analysis. As we have seen, the effect is due to a decrease in IBD sharing. A contrario, an error process which would increase IBD sharing would produce opposite results. The true error processes involved in practice are complicated mixtures of the models alluded to here. In our experience however, it seems that processes which lower IBD sharing are predominant. Because genotyping error tends to decrease the estimated number of alleles shared IBD, the effect on evidence for linkage is opposite in EC (reduced power) and ED (increased type I error) designs, it can be dramatic in typical designs and paradoxically less severe for more extreme ascertainment schemes. By analogy, for a dichotomous trait, this means that the effect of genotyping error is less severe in ASP designs for rare diseases than for common diseases. Remarkably, in designs combining both ED and EC pairs like the I (or EDAC designs), the competing effects of genotyping error tend to cancel each other out. We have considered here only three types of basic selection schemes however the approach can be straightforwardly applied to any arbitrary selection scheme. Under the widely accepted variance components model, the important quantity which determines bias, type I error and power is $A=\bar{C}/\bar{C^{2}}$ and it can be easily estimated by Monte Carlo simulations. Note that the bias is proportional to the error rate so that Equation (4) can easily be adapted to different error rates than those considered in Table 2.

Our study used an idealized model where IBD information is assumed to be complete. In practice, IBD is uncertain and it is inferred using marker data and multipoint algorithms as implemented in publicly available software [16,17], the general effect is to shrink the IBD estimate $\hat{\pi }$ towards 0.5. The linkage regression (2) is changed into $E(\hat{\pi }-\frac{1}{2}|x,\gamma ,\epsilon )≃{\mathrm{var}}_{0}(\hat{\pi })\gamma \;C(x,\rho )$ where ${\mathrm{var}}_{0}(\hat{\pi })<\frac{1}{8}$ can be either estimated from the data or by simulations. The effect of genotyping error is again mediated via the shift of the intercept in this regression but no general formula can be obtained because it depends in a very complex manner on the whole marker map configuration. Nevertheless, we can quantify this shift under realistic scenarios and compare it to its theoretical value when IBD information is complete. We simulated two different marker maps in 1 million sib pairs without parents and quantified by how much IBD sharing was reduced on average under the *population frequency error model *(error rate = 0.01). The microsatellites map (MS) had 13 equi-frequent ten-allele markers (heterozygozity = 90%) located 10 cM apart (spanning the 0–120 cM chromosomal region) and the SNP map had 41 equi-frequent SNPs (heterozygozity = 50%) spanning the 50–70 cM chromosomal region (this smaller region was chosen to keep simulation time acceptable). The resulting average reduction in IBD sharing for an error rate of 0.01 was measured every 2 cM in the 50–70 cM region, it ranged from 0.4974 to 0.4976 in the MS map and from 0.4945 to 0.4955 in the SNP map. For these two maps which mimic the two most widespread genotyping paradigms nowadays, those simulations confirm results derived under the complete marker information assumption with a reduction in IBD sharing from 0.5 to 0.5 – 0.01/4 = 0.4975. Our results therefore appear to be applicable to real-life situations where IBD information is incomplete.

The genomic-control strategy that we have proposed, although triggered by the specific issue of genotyping error, potentially offers a general robust method for carrying out linkage analysis. It is nonetheless important to recognize its limitations. Firstly, if the trait is highly polygenic with contributing genes scattered across the genome, the high correlation between linkage positions will make it impossible to estimate the IBD sharing at null positions. The genomic control strategy should therefore only be considered with oligogenic traits. Secondly, the concept of genomic control relies on the assumption that the genotyping error rates are similar across markers. For markers with a similar degree of polymorphism (number of alleles and frequencies), this assumption might be acceptable. In a multipoint setting, an additional assumption required to ensure the validity of a genomic control strategy is that inter-marker distances be approximately equal. With microsatellite markers, both these assumptions might fail resulting in differences in the IBD sharing reduction across markers. The 'regression-based linkage testing' view allows one to qualitatively assess how deviation from these assumptions will impact linkage testing. For example, in ASP or EC designs, wrongly assuming that IBD is uniformly reduced across markers will result in inflated type I error at marker positions with low genotyping error rate compared to other markers. The advent of SNP chips in linkage mapping holds the promise of regular marker maps with less variable information content than in classical microsatellites maps [2,3]. The many SNPs used are likely to be subject to similar genotyping error processes, this makes the critical assumption of the genomic control strategy all the more plausible. Alternatives to this genomic-control strategy are possible and they also consist in constraining the linkage regression through a new origin as in the ad-hoc method, the estimation procedure can be adapted to suit particular circumstances. Firstly, in random samples, the assumption regarding exchangeability of positions might be relaxed. Indeed, the reduction in IBD sharing at each position may be used as estimates of the position-specific intercepts (a study sufficiently powered to detect linkage in random samples should have a huge sample size which would ensure sufficient precision of the position-specific intercepts). However, it must be stressed that the advantage of using a genomic control in random samples is limited because the impact of genotyping error is small in such designs. Secondly, one could use previous lab data to estimate by how much IBD sharing deviates from its expected value, this could also be done at each position separately provided sufficient data are available. In practice, such data might not be available or they might not trustfully reflect current error mechanisms.

Elston et al. [18] have pointed out that the implicit assumption made in ASP designs, that randomly sampled sib pairs share half of their alleles IBD, might not hold in practice and have argued for including discordant pairs in such studies. The genomic control approach suggested here may be an alternative solution to this issue. Finally we note that, although we have only considered designs involving sib pairs, the approach naturally extends to other types of relative pairs.

## Conclusion

Under realistic genotyping error scenarios, power losses observed in extremely concordant designs are modest but the effect on type I error in extremely discordant designs can be dramatic. Our analytic approach provides some understanding of the differences in influence of genotyping errors across study designs. The advent of SNP arrays does not eliminate the impact of genotyping errors but it makes genomic control a feasible option with the potential to deliver more robust inference in linkage analysis data subject to genotyping errors or other mechanisms distorting the IBD signal.

## Abbreviations

ASP: affected sib pair; EC: extremely concordant; ED: extremely discordant; EDAC: extremely concordant and extremely discordant; IBD: identical-by-descent; QTL: quantitative trait locus; SNP: single-nucleotide-polymorphism.

## Authors' contributions

JJPL participated in the method development, carried out the simulations summarized in Table 1, drafted and finalized the manuscript. HP participated in method development and in drafting the manuscript. JJH-D and HCvH both participated in method development. All authors read and approved the final manuscript.
